# *Borrelia* sp. phylogenetically different from Lyme disease- and relapsing fever-related *Borrelia* spp. in *Amblyomma varanense* from *Python reticulatus*

**DOI:** 10.1186/s13071-016-1629-8

**Published:** 2016-06-24

**Authors:** Wachareeporn Trinachartvanit, Supanee Hirunkanokpun, Ronnayuth Sudsangiem, Wanwisa Lijuan, Duangjai Boonkusol, Visut Baimai, Arunee Ahantarig

**Affiliations:** Biodiversity Research Cluster, Department of Biology, Faculty of Science, Mahidol University, Rama 6 Road, Bangkok, 10400 Thailand; Center of Excellence for Vectors and Vector-Borne Diseases, Faculty of Science, Mahidol University at Salaya, Phutthamonthon 4 Road, Nakhon Pathom, 73170 Thailand; Department of Biology, Faculty of Science, Ramkhamhaeng University, Ramkhamhaeng Road, Bangkok, 10240 Thailand; Department of Science Education, Faculty of Science and Technology, Thepsatri Rajabhat University, Maung, Lopburi 15000 Thailand; Department of Biology, Faculty of Science and Technology, Thepsatri Rajabhat University, Maung, Lopburi 15000 Thailand

**Keywords:** *Borrelia*, *Amblyomma varanense*, *Amblyomma pattoni*, Tick, Thailand

## Abstract

**Background:**

Species of the genus *Borrelia* are causative agents of Lyme disease and relapsing fever. Lyme disease is the most commonly reported vector-borne disease in the northern hemisphere. However, in some parts of the world Lyme borreliosis and relapsing fever may be caused by novel *Borrelia* genotypes. Herein, we report the presence of a *Borrelia* sp. in an *Amblyomma varanense* collected from *Python reticulatus*.

**Methods:**

Ticks were collected from snakes, identified to species level and examined by PCR for the presence of *Borrelia* spp*. flaB* and *16S* rRNA genes*.* Phylogenetic trees were constructed using the neighbour-joining method.

**Results:**

Three *A. varanense* ticks collected from *P. reticulatus* were positive for a unique *Borrelia* sp., which was phylogenetically divergent from both Lyme disease- and relapsing fever-associated *Borrelia* spp.

**Conclusion:**

The results of this study suggest for the first time that there is a *Borrelia* sp. in *A. varanense* tick in the snake *P. reticulatus* that might be novel.

## Background

Tick infestation in snakes occurs worldwide and involves the following species: *Amblyomma gervaisi* in the northern region of western Ghats in India [1], *Rhipicephalus sanguineus* (*sensu lato*) in Malaysia [2], *Amblyomma varanense* and *Amblyomma helvolum* in Thailand [3, 4] and *Amblyomma hydrosauri* in Australia [5]. Hirunkanokpun et al. [6] detected several bacterial species in the national parks of Thailand, but no *Borrelia* spp. were found. The aim of this study was to determine the presence of *Borrelia* spp. within *Amblyomma* spp. ticks collected from five snake species. In addition, phylogenetic analyses of *Borrelia* spp. are also presented.

## Methods

### Tick collection and identification

Tick collection from snakes was performed in February 2014 in Lopburi Province, Thailand (14°48′1.61″N, 100°38′10.75″E). We observed snake scales to identify partial protrusions of tick bodies outside of the scales. Ticks were collected from the skin beneath the scales using forceps. Ticks were identified according to their morphology using standard taxonomic keys [7–11].

### DNA extraction and amplification

The ticks were washed individually and homogenised in 200 μl of 10× PBS solution. DNA extraction was performed using the QIAamp® DNA Blood Mini Kit (Qiagen, Hilden, Germany). Oligonucleotide primer pairs FLA1-FLA2 (BflaPAD 5′-GAT CA(G/A) GC(T/A) CAA (C/T)A TAA CCA(A/T) ATG CA-3′; BflaPBU, nest- 5′-GCT GAA GAG CTT GGA ATG CAA CC-3′; BflaPCR, nest-5′-TGA TCA GTT ATC ATT CTA ATA GCA-3′; BflaPDU 5′-AGA TTC AAG TCT GTT TTG GAA AGC-3′) and 16S rDNA (16SF1 5′-ATA ACG AAG AGT TTG ATC CTG GC-3′; 16SR 5′-CAG CCG CAC TTT CCA GTA CG/3′) were used in this study to identify target *Borrelia* DNA in the ticks [7, 8]. The positive PCR products from tick samples were purified using a High Pure PCR product purification kit (Roche, Basel, Switzerland). Sequencing reactions were performed with BigDye Terminator v3.1 Cycle Sequencing Kits (Applied BioSystems, Waltham, Massachusetts, USA) based on the fluorescent-label terminator method. Sequencing products were analyzed using a Genetic Analyzer 3730XL automated DNA sequencing system (Applied BioSystems, Waltham, Massachusetts, USA).

### Phylogenetic analysis

Phylogenetic trees were constructed using the neighbour-joining method (PAUP 4.0b1) [12]. DNA gaps or missing data were excluded from the analyses. Confidence values for individual branches of the resulting tree were determined by bootstrap analysis with 1000 replicates.

## Results and discussion

**Fig. 1:**
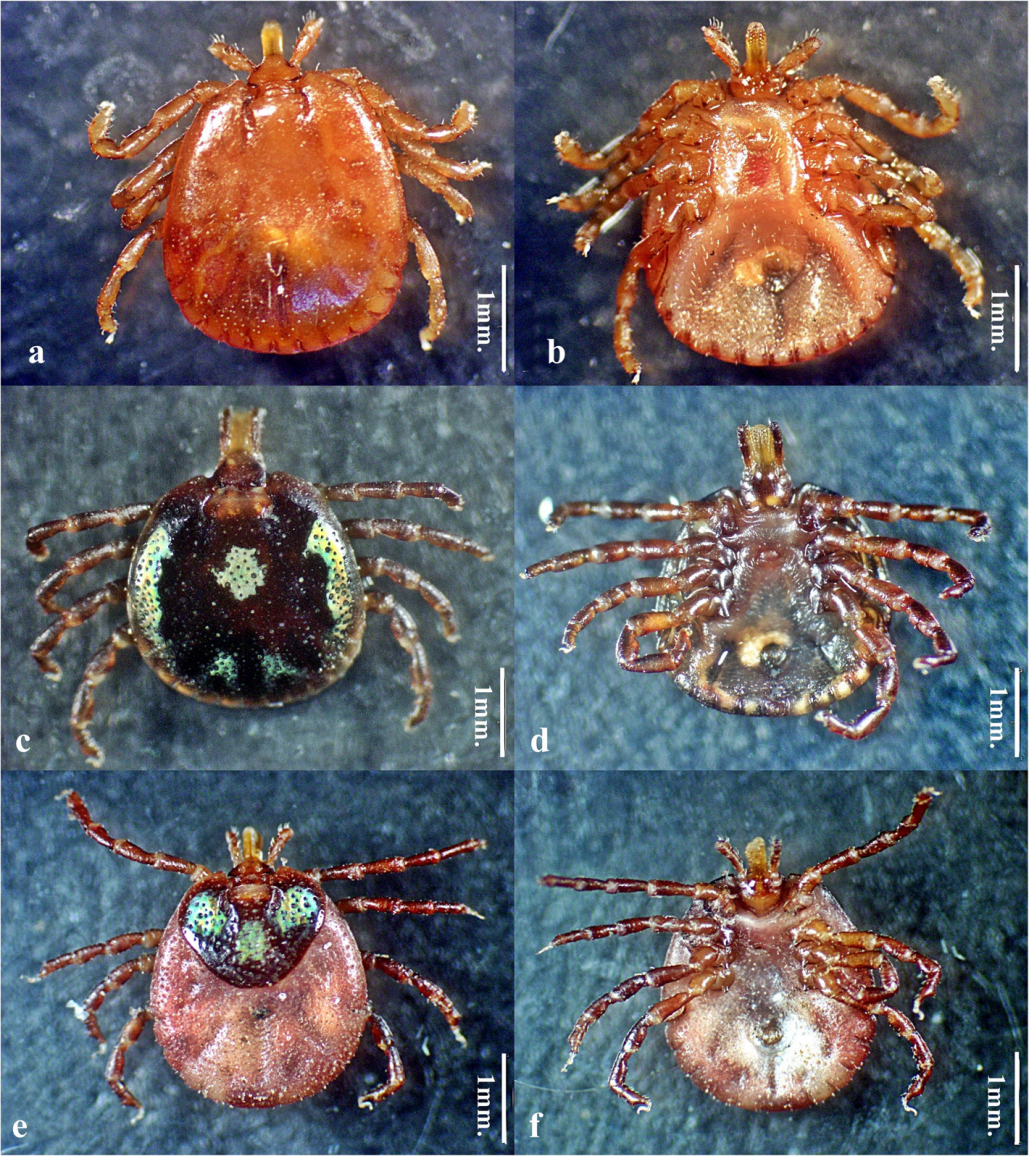
Pictures of ticks identified in this study. **a** *Amblyomma pattoni*, male, dorsal view. **b** *Amblyomma pattoni*, male ventral view. **c** *Amblyomma varanense*, male, dorsal view. **d** *Amblyomma varanense*, male, ventral view. **e** *Amblyomma varanense*, female, dorsal view. **f** *Amblyomma varanense*, female, ventral view. *Scale-bars*: 1 mm

Ticks were collected from the following five snake species: *Python reticulatus*, *Ophiophagus hannah*, *Ptyas korros*, *Naja kaouthia* and *Elaphe radiata*. Four *Amblyomma varanense* ticks (three males and one female) were collected from one *P. reticulatus.* One *A. varanense* tick (one female) and two *Amblyomma pattoni* (males) were collected from *O. hannah.* Two *A. pattoni* (males) were collected from *N. kaouthia.* In addition*,* two *A. pattoni* males were collected from *E. radiata*. Finally*,* one *A. varanense* tick (male) was collected from *E. radiata.* Tick species reported in this study have hypostomal dentition 3/3. The male of *A. pattoni* has coxa I with an inconspicuous internal spur, which is sometimes fused with the more prominent external spur, and cervical pits are comma-shaped. The male of *A. varanense* has coxa I with the external spur noticeably longer than the internal and the female has coxa I with the internal spur smaller than the external spur, but always separated from the latter (Fig. 1).

A total of 12 ticks was collected from snakes and examined by PCR for the presence of the *Borrelia* spp*.* genes*.* Of these, three ticks, all identified as *A. varanense* isolated from *P. reticulatus*, were positive for *Borrelia* spp. No *Borrelia* spp. were detected in *A. pattoni* or in *A. varanense* collected from other snake species.

*Borrelia* sp. DNA sequences were compared with sequences in the NCBI GenBank database by nucleotide BLAST. The *16S* rRNA gene sequence of this *Borrelia* sp. is 100 % identical (1449/1449 bp) to *Borrelia* sp. BF16 (GenBank: AB473538) and was submitted to GenBank and assigned as KU497718 (*Borrelia* sp. in *Amblyomma varanense* from *Python reticulatus*). The amplified PCR product of the *flaB* gene for flagellin was approximately 384 bp. A sequence analysis of the *flaB* gene for flagellin from the *Borrelia-*positive ticks revealed that this gene sequence is 99 % similar (294/296 bp) to a sequence for *Borrelia* sp. BF16 *flaB* gene for flagellin (isolate BF16; GenBank: AB473488). The 384 bp *Borrelia flaB* consensus sequence was submitted to GenBank and assigned as *Borrelia* sp. KT758064. By contrast, this sequence was only 87 % similar (337/387 bp) to the following: the *B. turcica flaB* gene for flagellin (strain IST4; GenBank: AB109244); *B. turcica* IST7 flagellin gene (GenBank: KF422815) (336/387 bp); and *Borrelia* sp. tAG66 M *flaB* gene for flagellin (GenBank: AB529322) (336/387 bp).

**Fig. 2:**
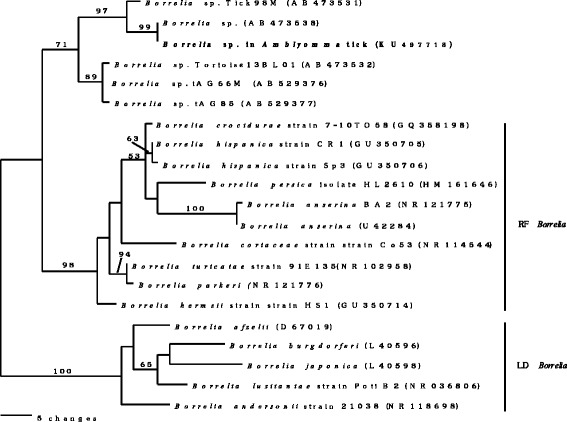
Neighbour-joining phylogenetic analysis of *Borrelia 16S* rRNA gene sequences including the newly-generated KU497718 for *Borrelia* sp. in *Amblyomma varanense* from *Python reticulatus*

**Fig. 3:**
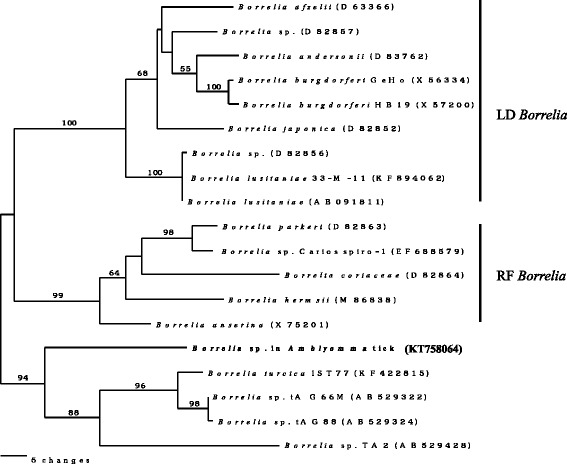
Neighbour-joining phylogenetic analysis of *Borrelia flaB* gene sequences including the newly-generated KT758064 for *Borrelia* sp. in *Amblyomma varanense* from *Python reticulatus*

Phylogenetic trees were constructed using the neighbour-joining method (PAUP 4.0b1) [12]. Individual branch confidence values were determined by bootstrap analysis with 1000 pseudoreplicates (Figs. 2 and 3). The phylogenetic trees for both genes of *Borrelia* spp. inferred from the complete *16S* rRNA gene sequences (Fig. 2) and partial sequences of the *flaB* genes (Fig. 3) indicated that *Borrelia* sp. from the present study is related to *Borrelia* sp. (GenBank: AB473538) from reptiles and to *B. turcica* IST7 (GenBank: KF422815) flagellin gene, respectively, but belongs in a different group from *Borrelia burgdorferi*. The phylogenetic relationships among relapsing fever-associated *Borrelia* spp. and Lyme disease-associated *Borrelia* spp. were reported previously using rrs and *16S* rDNA [13, 14]. The *flaB* and *16S* rRNA gene sequences of *Borrelia* sp. of *A. varanense* from *P. reticulatus* isolated in this study, formed a separate branching root from both Lyme disease-associated *Borrelia* species and relapsing fever-associated *Borrelia* species.

## Conclusion

Our findings suggest that *Borrelia* sp. in *A. varanense* from *P. reticulatus* might be novel and phylogenetically divergent from both Lyme disease- and relapsing fever-associated *Borrelia* species.
